# Extensive and recurrent infection caused by *Medicopsis romeroi* in two immunocompromised patients

**DOI:** 10.1016/j.mmcr.2025.100706

**Published:** 2025-05-08

**Authors:** Lotte Keikes, Marit S. van Sandwijk, Evert-Jan Kooi, Marieke Gittelbauer, Karin van Dijk

**Affiliations:** a Amsterdam University Medical Center, Department of Internal Medicine, Division of Infectious Diseases, Meibergdreef, 1105, AZ, Amsterdam, Netherlands; b Amsterdam University Medical Center, Department of Internal Medicine, Division of Nephrology, Meibergdreef, 1105, AZ, Amsterdam, Netherlands; c Amsterdam University Medical Center, Department of Pathology, Meibergdreef, 1105, AZ, Amsterdam, Netherlands; d Amsterdam University Medical Center, Department of Medical Microbiology, Division of Medical Mycology, Meibergdreef, 1105, AZ, Amsterdam, Netherlands

**Keywords:** *Medicopsis romeroi*, Dematiaceous fungi, Fungal infection, Phaeohyphomycosis, Immunocompromised patients, Solid organ transplant recipients

## Abstract

*Medicopsis romeroi*, a rare brown-pigmented mold, is one of the causes of phaeohyphomycosis, a (sub)cutaneous or soft tissue fungal infection with formation of nodules, cysts or abscesses. Mainly immunocompromised patients are affected, who may experience a wider spectrum of disease with involvement of other tissues, such as the bones or the sinuses. No specific treatment recommendations are available, but surgical excision appears to be the mainstay of treatment, combined with (long-term) antifungal therapy. In this case series, we describe two immunocompromised patients with extensive and persistent skin lesions caused by *M*. *romeroi*, and clinical practice recommendations for optimal treatment.

## 1 Introduction

*Medicopsis romeroi*, also known as *Pyrenochaeta romeroi*, belongs to the group of dematiaceous fungi, brown-pigmented molds characterized by melanin pigments in their cell walls. It is a heterogeneous group of fungi causing phaeohyphomycosis, a (sub)cutaneous or soft tissue infection consisting of skin nodules, cysts and the formation of abscesses, usually occurring on the extremities [1]. Immunocompromised hosts are mainly affected, in particular solid organ transplant recipients, but patients on steroids, patients with malignancies and patients with diabetes and poor glycemic control are also at risk [1]. Although rare, cases of immunocompetent patients have been described [2]. In immunocompromised hosts, other tissues than the skin such as the sinuses, eyes, joints and bones may be involved [1].

*M. romeroi* is present in soil, wood or decaying plants and prevalent in mainly tropical or subtropical environments [3]. It invades the skin or mucous membranes such as the cornea through minor injuries or scratches. The lesions may appear many years after the initial occurrence of the traumatic inoculation, in particular after the start of immunosuppressive therapy [4].

Previous literature of infections due to *M. romeroi* is scarce. Some case reports and small case series have been published [5,6]. This case series describes two immunocompromised patients with extensive and recurrent infection caused by *M. romeroi*, and provides best practice recommendations for diagnostics and optimal treatment.

## 2 Case presentation 1

A 61-year-old male patient of Ghanaian descent presented with an abscess of the third finger of the left hand (day 0) (Fig. 1a). He had been living in the Netherlands for 44 years and his last visit to Ghana was more than 20 years ago. He was affected with end-stage kidney failure due to uncontrolled hypertension and underwent an uncomplicated kidney transplantation eight months before presentation. His immunosuppressive therapy consisted of tacrolimus, mycophenolate mofetil and prednisolone.

**Fig. 1a:**
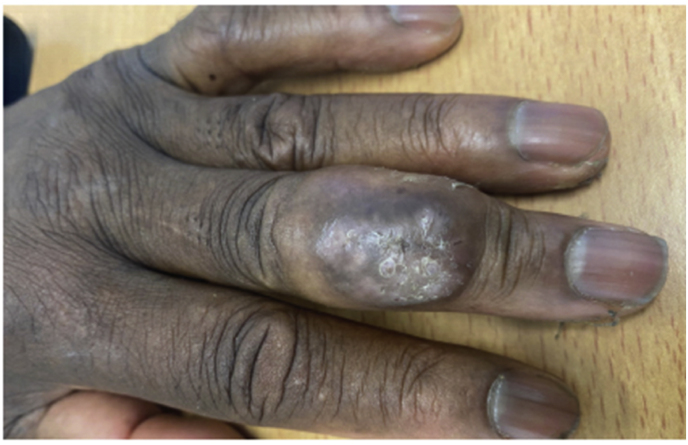
Case 1: Initial lesion on the third finger of the left hand (day 0).

He underwent five times incision and drainage (day 0, day +31, day +48, day +69 and day +100), followed by four times broad-spectrum antibiotics (day +31, day +48, day +69 and day +100). A total of three wound smears (day 0, day +31 and day +100) were sent for bacterial culture, but all turned out to be negative. The mycophenolate mofetil was stopped approximately three months after presentation (day +98) to improve his immune status. Eight (day +249) and twelve months (day +376) after the initial presentation, biopsies were taken. The first biopsy was solely evaluated for gout crystals at the request of the rheumatologist and the second biopsy turned out negative for bacteria, molds, actinomycosis and (non-)tuberculous mycobacteria.

A year and one month (day +391) after presentation, the patient experienced progression of the initial lesion and a second more proximal lesion of the same finger appeared (Fig. 1b). In addition, a subsequent third lesion with a similar phenotype had developed at the left elbow (day +391) (Fig. 1c). A biopsy was taken of the lesion of the elbow (day +411), which was sent for fungal culture. The Blancophor staining was positive for fungal hyphae, and a grey to black mold grew on the Sabouraud dextrose agar after 3 days (day +425) (incubation on day +422) (Fig. 2a, Fig. 2b, Fig. 2ca, b, 2c). A Gram and perm-blue stain are shown in Fig. 3, Fig. 4. DNA sequencing of the mold demonstrated *M. romeroi*. Antifungal susceptibility testing was performed and resulted in low minimal inhibitory concentrations (MICs) for posaconazole (0.5 mg/L), voriconazole (1 mg/L), isavuconazole (0.25 mg/L) and anidulafungin (0.008 mg/L) and high MICs for amphotericin-B (4 mg/L) and itraconazole (>8 mg/L). No clinical breakpoints are known for *M. romeroi.* Antifungal therapy, consisting of voriconazole (200 mg twice daily) was started (day +433). Therapeutic drug monitoring (TDM) was performed with a target value between 2 and 6 mg/L. Three weeks after starting antifungal therapy, the patient underwent surgical excision of both lesions of the finger (day +454) (Fig. 5) and the elbow. The histopathological images with typical multiple septate hyphae are shown in Fig. 6. The patient was treated with voriconazole for a total of seven months (stopped on day +642), with no signs of recurrence to date.

**Fig. 1b:**
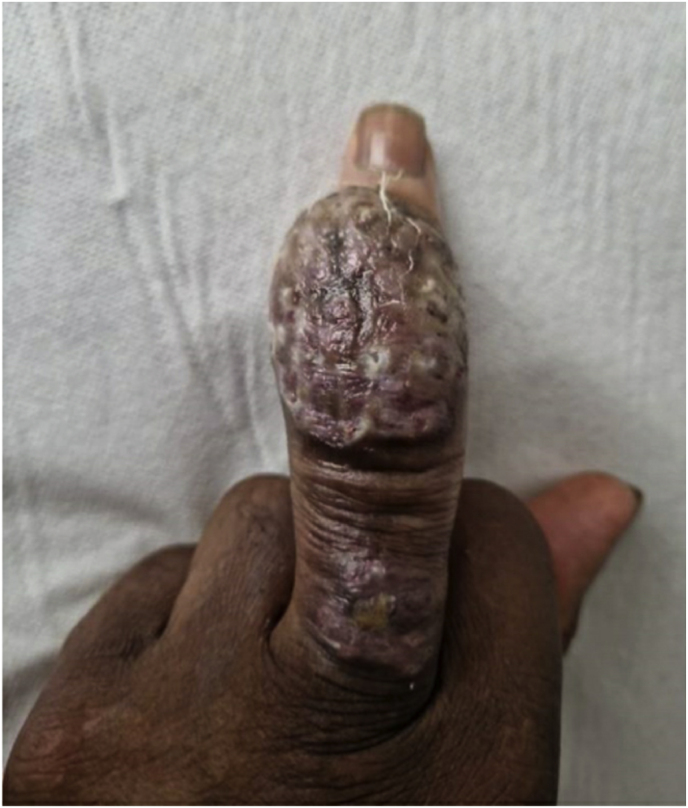
Case 1: Progression of the initial lesion and a second more proximal lesion on same finger (day +391).

**Fig. 1c:**
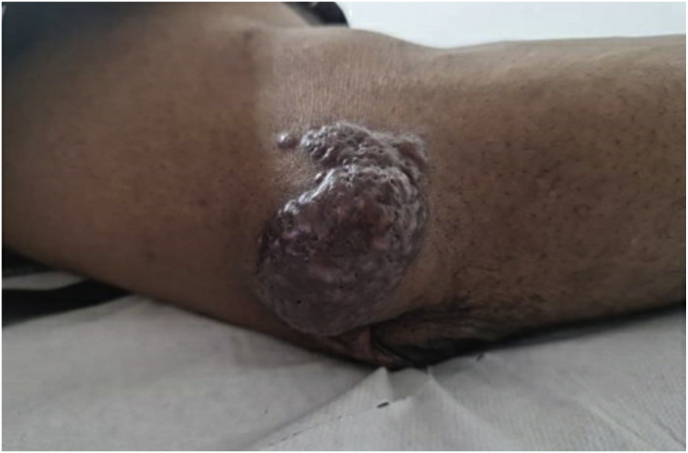
Case 1: Third lesion with similar phenotype on the left elbow (day +391).

**Fig. 2a:**
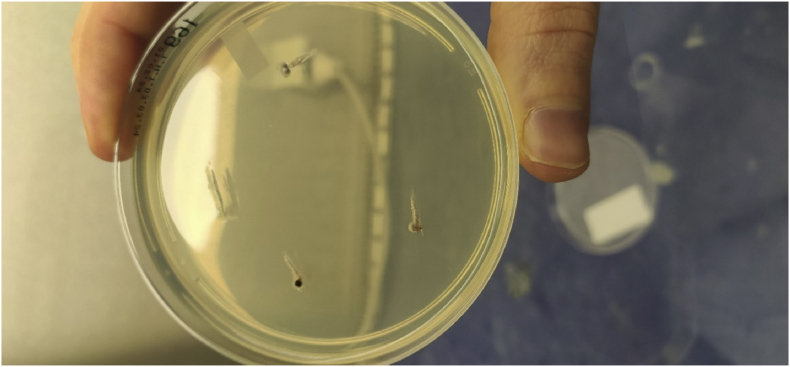
Growth of *M. romeroi* on Sabouraud glucose agar (+3 days after incubation; day +425).

**Fig. 2b:**
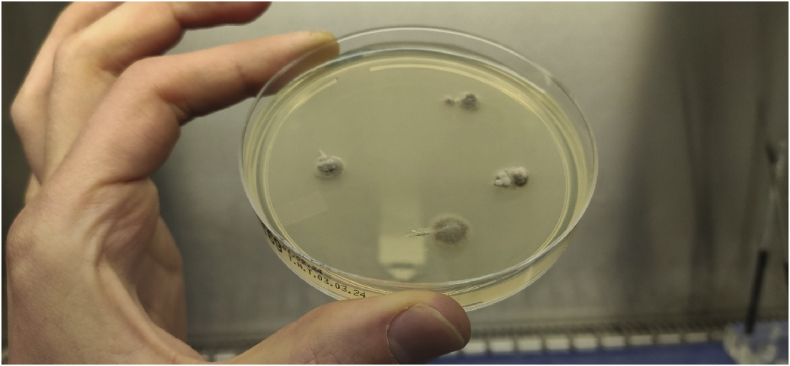
Growth of *M. romeroi* on Sabouraud glucose agar (+8 days after incubation; day +430).

**Fig. 2c:**
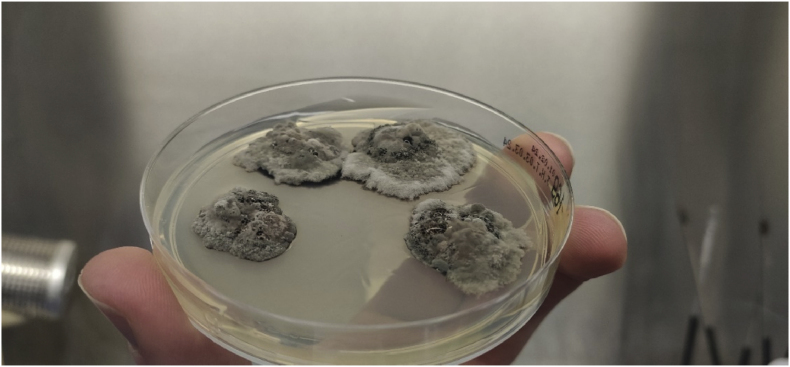
Growth of *M. romeroi* on Sabouraud glucose agar (+36 days after incubation; day +458).

**Fig. 3:**
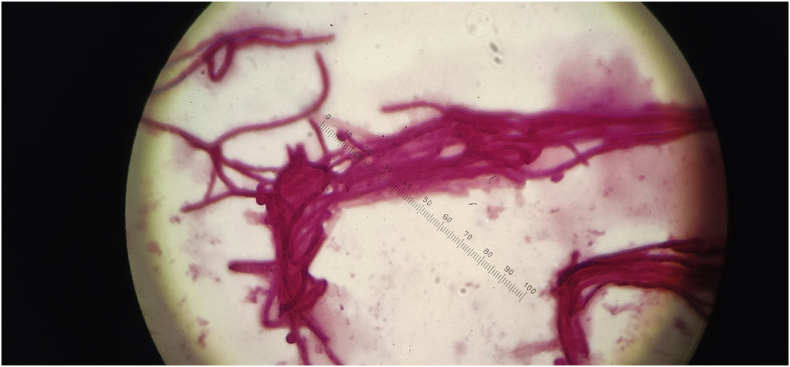
Grain stain showing fungal hyphae of *M. romeroi*.

**Fig. 4:**
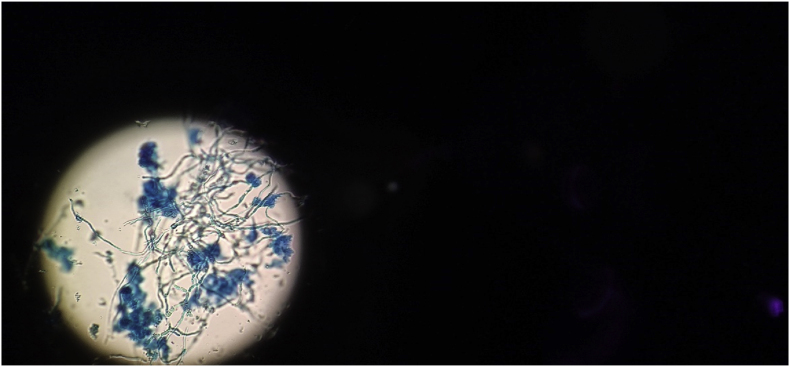
Perm blue stain showing fungal hyphae of *M. romeroi*.

**Fig. 5:**
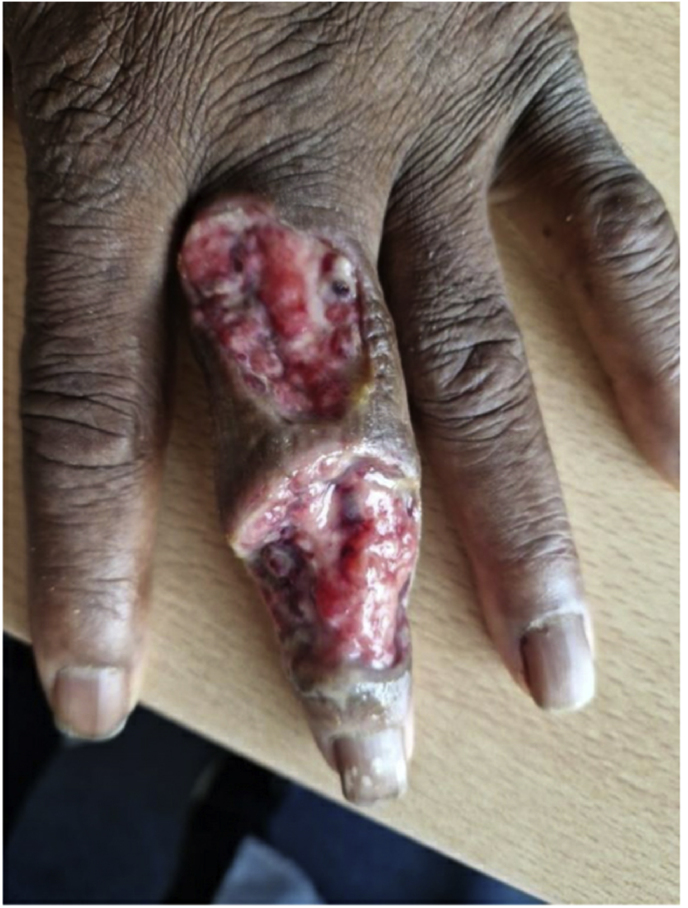
Case 1: Evaluation after surgical excision of both lesions (day +454).

**Fig. 6:**
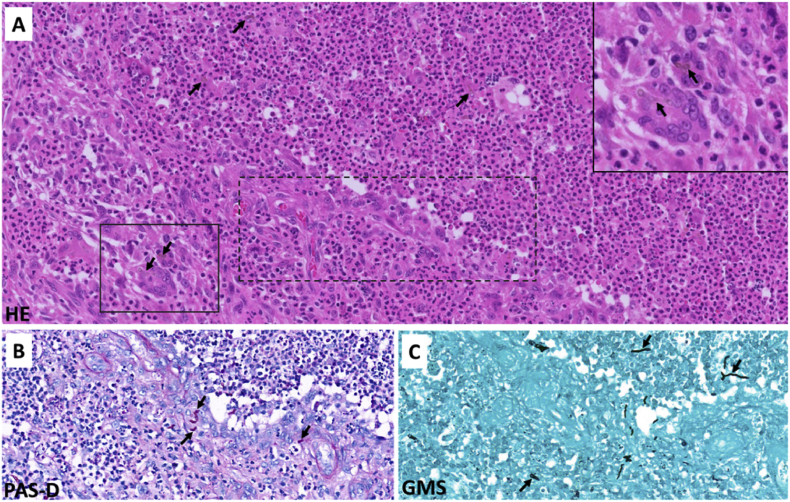
Histological images of pigmented hyphal cutaneous infection (day +454). A. Hematoxylin and eosin (HE)-stained section showing dense neutrophilic infiltration with several brown-pigmented, septate hyphae within the dermis. Inset: High-power view of the uninterrupted boxed area highlights septate hyphae consistent with fungal elements compatible with Phaeohyphomycosis. B and C. Periodic acid–Schiff (PAS-D) and Grocott's methenamine silver stain (GMS) stains, respectively, corresponding to the interrupted boxed area in panel A, demonstrate multiple septate hyphae.

## 3 Case presentation 2

A 49-year-old male patient of Ghanaian descent presented with a fast-growing swelling on the right forearm (day 0). He had been living in the Netherlands for 27 years. In the years before presentation, he visited Ghana regularly, most recently a month ago. He underwent a kidney transplantation two years before presentation due to end-stage kidney failure as a result of type 2 diabetes and uncontrolled hypertension. His immunosuppressive therapy consisted of tacrolimus, mycophenolate mofetil and prednisolone.

**Fig. 7:**
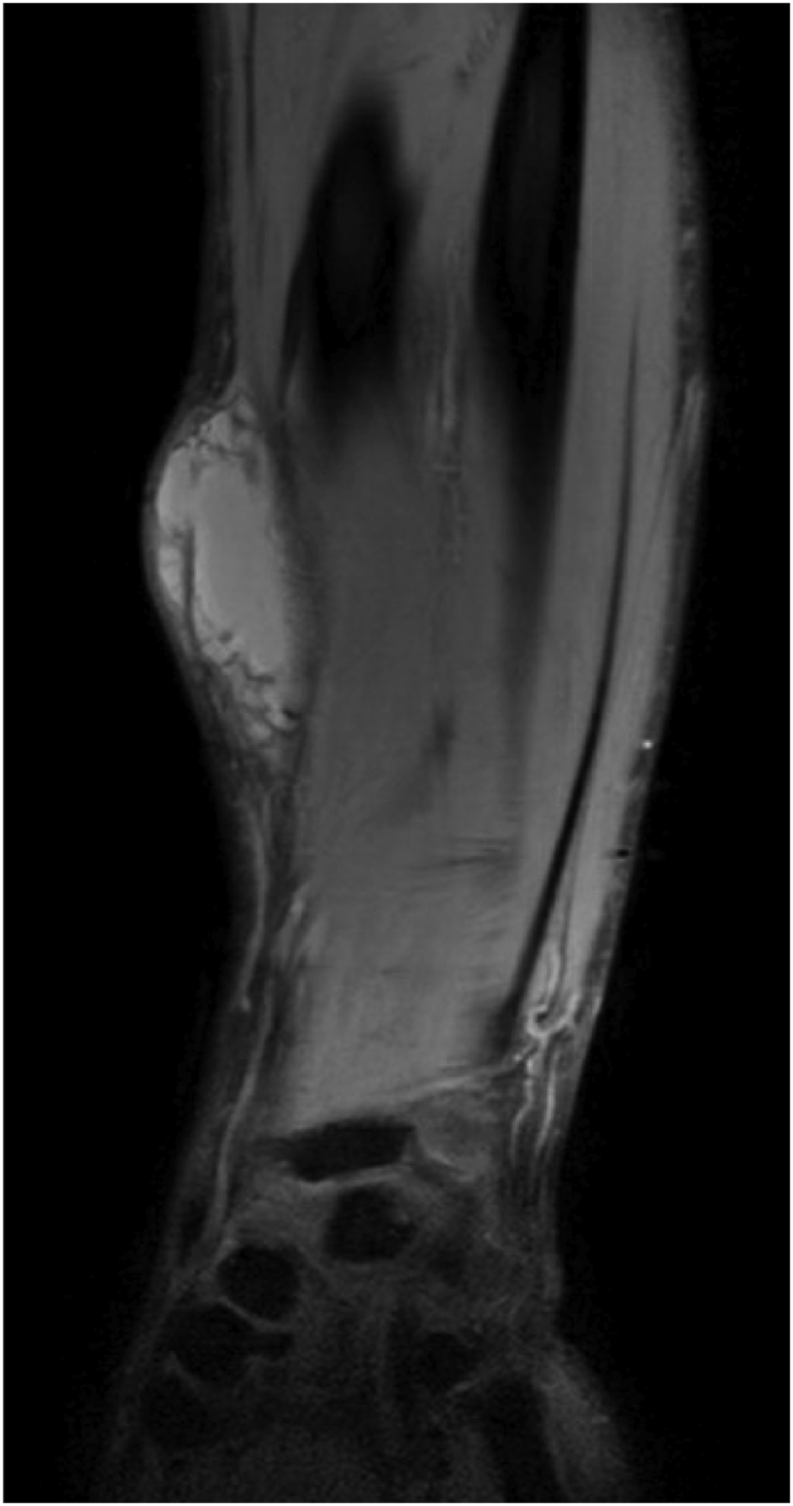
Case 2: Lesion of the right forearm on MRI (day +3).

Initially, the patient was referred to the surgeon, who requested a magnetic resonance imaging (MRI) (day 3+), which showed a lesion (5 by 5 by 2 cm) of unknown origin (Fig. 7). A week later (day +10), an ultrasound-guided biopsy was taken and the pus of the lesion was completely aspirated. A fungus grew in the biopsy culture and DNA sequencing demonstrated *M. romeroi*. Susceptibility testing for antifungal therapy could not be performed due to the lack of sporulation.

Initially, the lesion was solely treated by incision and drainage, but this resulted in rapid recurrence. Seven weeks after biopsy (day +62), the mycophenolate mofetil was interrupted and voriconazole (200 mg twice daily) was started. TDM was performed for voriconazole with a target level between 2 and 6 mg/L. Shortly after initiating antifungal therapy, the patient experienced an allergic reaction to voriconazole with facial swelling and a sore mouth, and antifungal therapy was switched to posaconazole (300 mg once daily) (day +83). TDM was performed with a target value higher than 1.5 mg/L.

**Fig. 8:**
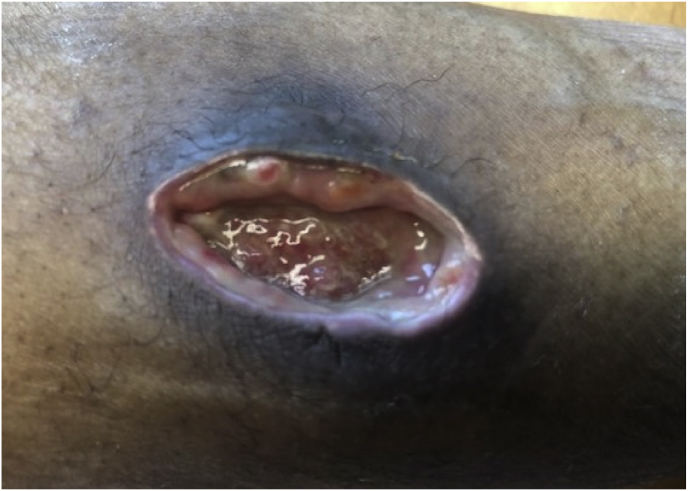
Case 2: Evaluation of the lesion after surgical drainage and excision (day +216).

**Fig. 9:**
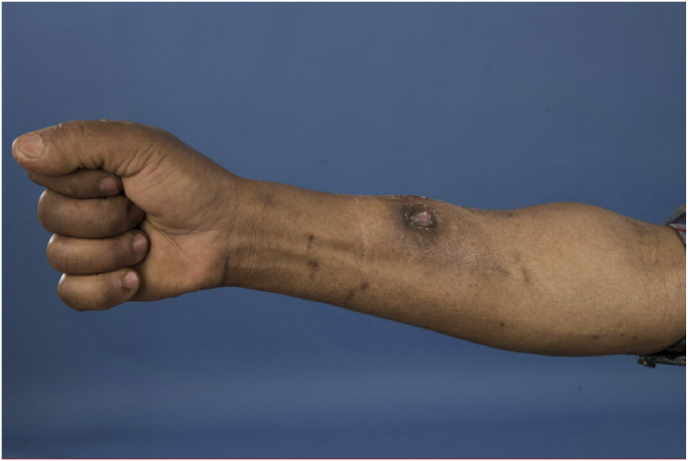
Case 2: Seven months after first surgical drainage and excision (day +413).

After three times of incision and drainage (day +70, day +107, day +174) and recurrence of the lesion, surgical drainage and excision was performed (Fig. 8, day +216). However, the postoperative course was suboptimal, necessitating two additional surgical incisions (day +300 and day +337), but the lesion continued to exhibit pustular discharge (day +413) (Fig. 9). Furthermore, new biopsies were taken, due to doubts about the diagnosis. However, the pathology of the biopsies continued to show a fungal abscess.

**Fig. 10:**
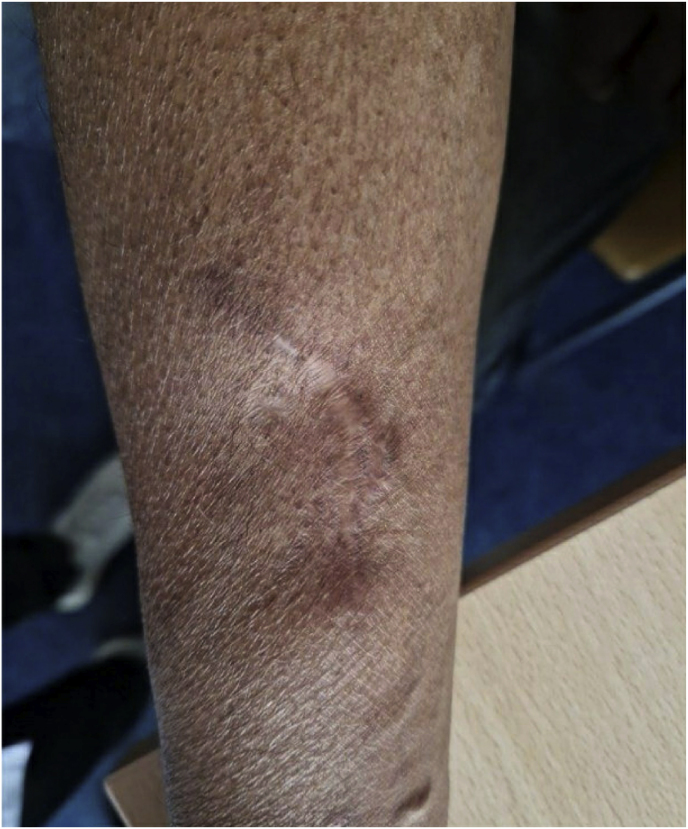
Case 2: No signs of recurrence to date (day + 1648).

Ultimately, one year and eleven months after the initial presentation (day +696), surgical debridement and excision of the lesion were performed, which resulted in complete remission. The fungal culture of the perioperative biopsies confirmed the persistence of *M. romeroi.* Antifungal therapy was discontinued approximately six weeks (day +743) after surgical intervention. Overall, the patient was treated with posaconazole for almost two years. To date, there are no signs of recurrence (day +1648) (Fig. 10).

## 4 Discussion

We describe two immunosuppressed patients with extensive and persistent disease manifestations of *M. romeroi*, highlighting the potential invasive character of this fungal infection, and although rare, the relevance of its recognition to improve clinical outcomes. The first case concerned a patient with multiple lesions of *M. romeroi*; the second case described extensive persistence of *M. romeroi* of the same location. In both cases, treatment was challenging and surgical excision, reduction of immunosuppressive therapy and (long-term) antifungal therapy were combined, although surgical excision seemed to be the mainstay to achieve complete remission.

Fungal infection caused by *M. romeroi* mainly presents with lesions on the extremities, which was also demonstrated in our case descriptions [4]. Both patients experienced multiple recurrences on the same location, which is described in previous literature [7]. The first patient was affected by a subsequent second, more proximal lesion of the same finger and a third distant lesion on the elbow. Although cases of patients with multiple lesions have been described, it remains an uncommon presentation of fungal infection caused by *M. romeroi* [5].

The diagnosis of *M. romeroi* can be challenging. An important variable in the diagnostic process is the clinician's consideration of other than usual causes of infection, including fungal infection in immunocompromised hosts. This is particularly important in patients who visited or grew up in subtropical of tropical environments, since lesions may appear months to years after the initial traumatic inoculation [4]. In the second case, biopsies were performed early in the diagnostic process and resulted in a prompt diagnosis. In the laboratory, the fungus may be difficult to identify due to its slow-growing character or if a concurrent infection occurs with a faster growing fungus resulting in overgrow [8].

Antifungal susceptibility testing of *M. romeroi* may be difficult due to the weak formation of fungal spores, and is therefore often not performed. In studies with successful susceptibility testing, low MICs have been documented for voriconazole and posaconazole, while high MICs were documented for fluconazole, ketoconazole, caspofungin and itraconazole [5,9,10]. Amphotericin-B and terbinafine had variable MICs [5,11].

Evidence-based treatment recommendations of *M. romeroi* are not available and different treatment strategies have been described [3,4]. In some cases, solely surgical excision has been successful, or surgery combined with reduction of immunosuppressive agents [3,12]. Also curative treatment with only antifungals has been described, although follow-up is not always properly documented [3,13]. In some cases, initial antifungal therapy was insufficient and surgical excision was still required at a later stage [3,14]. The most successful outcomes seem to be achieved by combining surgery with antifungal therapy, with or without adjustment of immunosuppressive therapy [4,15]. However, cases of treatment failure combining both surgery and antifungal therapy have been described [7]. Based on our experience, incision and drainage instead of surgical excision is insufficient to achieve complete remission. This may be a result of the fungus growing into the edges of the abscess, resulting in regrowth after drainage alone. Therefore, we recommend surgical excision including the edges of the abscess [16]. The adjustment of immunosuppressive therapy may be pivotal and should be cautiously considered. In both cases presented, mycophenolate mofetil was interrupted without affecting transplant function or inducing graft rejection, and may have played an important role in cure and prevention of recurrence. In previous literature, the duration of antifungal therapy varied between several weeks to several years [3,4]. No treatment recommendations are available, but it seems logical to continue antifungal therapy at least until complete remission is achieved. Recent studies describe the use of imiquimod therapy in patients with chromoblastomycosis. In *M. romeroi,* this has not yet been described. However, this may be an option in patients with surgery and antifungal therapy resistant disease [17,18].

In conclusion, *M. romeroi* is a rare but relevant fungal pathogen, in particular in immunosuppressed patients who visited or grew up in (sub)tropical areas. Based on our experience and previous literature, we recommend to take a biopsy of the suspected lesion early in the diagnostic process and send it in for fungal culture to improve the chance of a prompt diagnosis. The optimal treatment regimen should combine surgical excision, careful consideration of immunosuppressive therapy adjustment and antifungal therapy, in order to achieve complete remission and prevent recurrence or dissemination of the infection.

## CRediT authorship contribution statement

**Lotte Keikes:** Writing – original draft, Visualization, Project administration, Methodology. **Marit S. van Sandwijk:** Writing – review & editing, Resources. **Evert-Jan Kooi:** Resources, Writing – review & editing. **Marieke Gittelbauer:** Resources, Investigation. **Karin van Dijk:** Writing – review & editing, Resources, Project administration, Conceptualization.

## Consent

Written informed consent was obtained from the patient or legal guardian(s) for publication of this case report and accompanying images. A copy of the written consent is available for review by the Editor-in-Chief of this journal on request.

## Funding source

This study is unfunded.

## Declaration of competing interest

*L. Keikes* declares that she has no conflict of interest.

*M.S. van Sandwijk* declares that she has no conflict of interest.

*E.J. Kooi* declares that he has no conflict of interest.

*M. Gittelbauer* declares that she has no conflict of interest.

*K. van Dijk* received a speaker's fee and an advisory board fee from 10.13039/100004319Pfizer and a speaker's fee and an advisory board fee from Gilead.
